# Silver nanoparticles-based green fluorescent probe for determination of Bosentan in pharmaceutical formulation and spiked plasma samples

**DOI:** 10.1186/s13065-026-01737-w

**Published:** 2026-02-21

**Authors:** Yossra A. Trabik, Rehem A. Ismail, Miriam F. Ayad, Lobna A. Hussein

**Affiliations:** Department of Pharmaceutical Analytical Chemistry, Faculty of Pharmacy, Ain Shams University, Abbassia, Cairo, 11566 Egypt

**Keywords:** Bosentan, Silver nanoparticles, Fluorescent probe, Analytical eco-scale, Agree software, Human plasma

## Abstract

In this work, a new, sensitive, precise, accurate, and eco-friendly spectrofluorometric method for the detection of Bosentan in human plasma and pharmaceutical dosage form was developed, using silver nanoparticles (AgNPs) as an efficient fluorescence sensing probe. The general concept used in this approach is based on collisional quenching of AgNPs fluorescence intensity. Over the concentration range of 0.02 to 0.12 µg/mL, the quenching impact of BOS on AgNPs increased as its concentration increased. The suggested method’s greenness was assessed using two metric systems: the Analytical Eco-scale (AES) with a score of 80 and the AGREE greenness assessment tool with a score of 0.68.

**Graphical Abstract:**

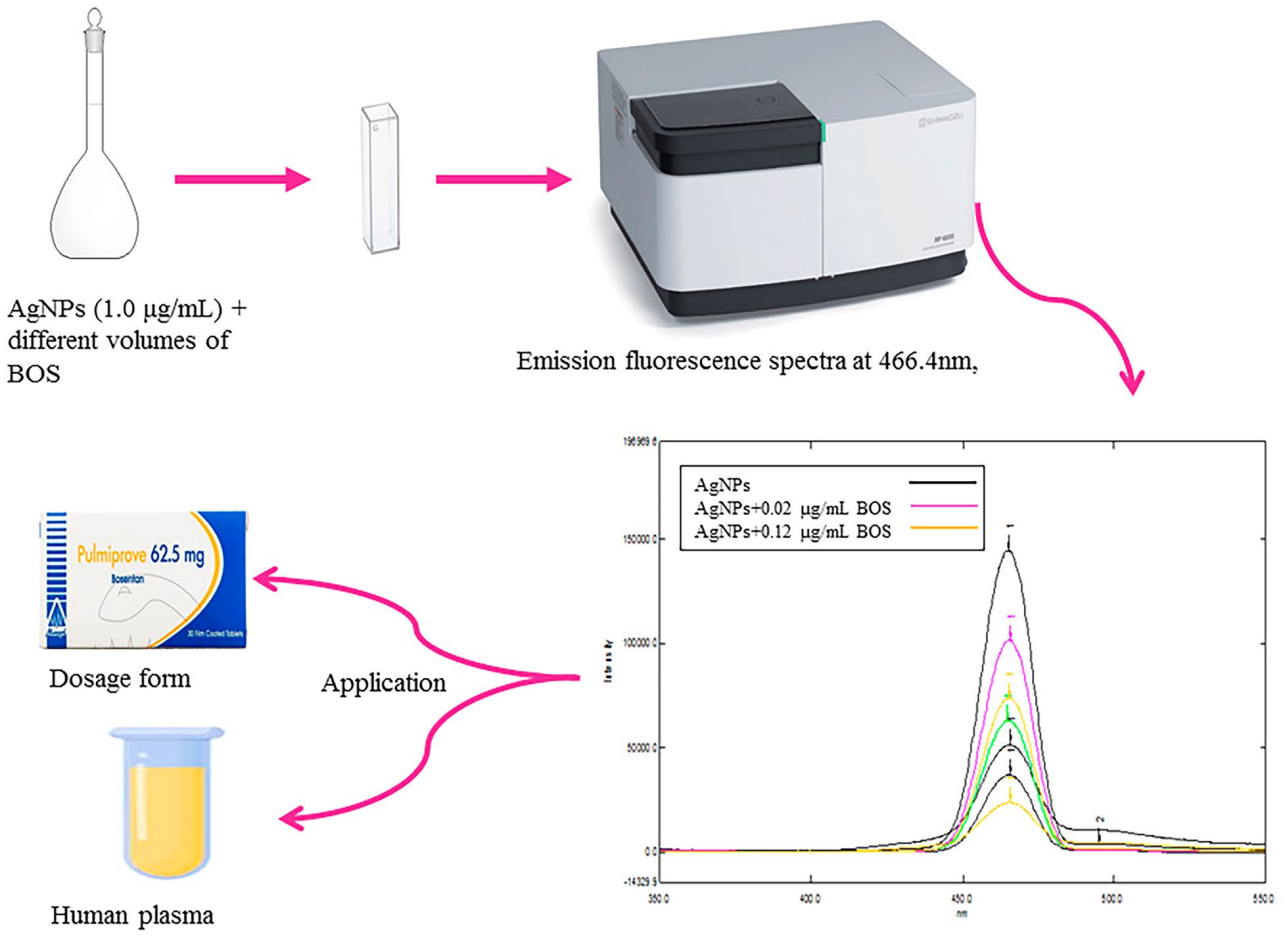

**Supplementary Information:**

The online version contains supplementary material available at 10.1186/s13065-026-01737-w.

## Introduction

More than 100 million people worldwide suffer from pulmonary hypertension (PH), a serious illness with several etiologies. This condition is characterized by an increase in mean pulmonary arterial pressure (mPAP) of ≥ 25 mmHg while at rest. PH progressively worsens and can be fatal due to several complications, such as blood clots in the lungs’ small arteries and heart failure. Endothelin receptor antagonists (ERAs), such as Bosentan, are crucial in the treatment of PH because they prevent the activity of endothelin, a vasoconstrictor that is abundantly expressed in PH patients [1].

**Fig. 1:**
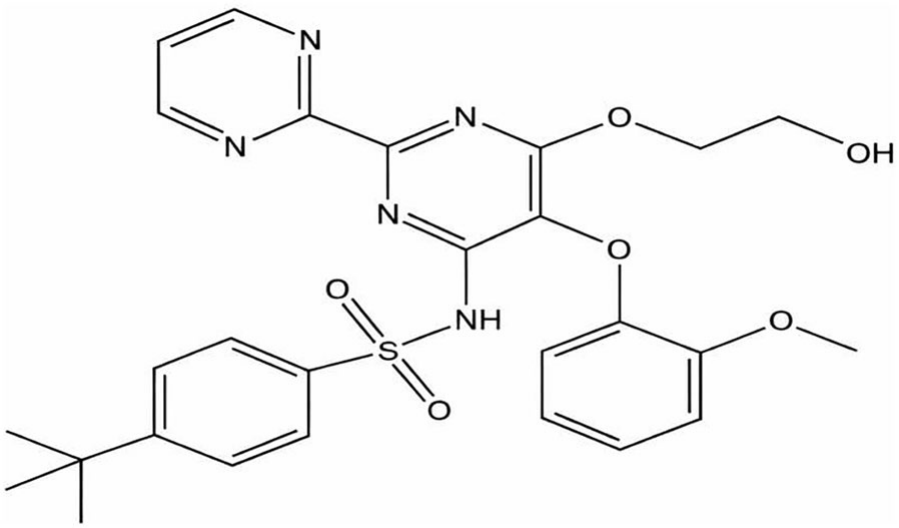
Chemical structure of Bosentan (drawn by chemibio draw ultra)

Bosentan (BOS) (4-tert-butyl-N-[6-(2-hydroxyethoxy)-5-(2-methoxyphenoxy)-2-(pyrimidin-2-yl) pyrimidin-4-yl] benzene sulfonamide) (Fig. 1), prevent the action of endothelin by blocking endothelin A and B receptor. The US Food and Drug Administration (FDA) authorized BOS as the first oral active medication to treat PH in 2001. It alleviates symptoms through vasodilation, antifibrotic, and antithrombotic effects particularly in those with WHO Class III or IV symptoms. BOS’s antiviral properties also make it a promising medical treatment for COVID-19, when combined with other authorized medications [2, 3].

Many analytical approaches, including chromatography, spectroscopy, and potentiometry, have been reported for BOS measurement in pharmaceutical dosage forms and biological fluids [4–9]. But as far as we are aware, no spectrofluorometric method has been documented for determining BOS. Spectrofluorimetric methods are generally simple, rapid, highly sensitive, require minimal costs, and are more eco-friendly than other methods [10]. The special benefits of fluorescent probes include their ease of use, high selectivity, fast response, and anti-interference capability. These benefits are essential for the food and pharmaceutical industries, as well as the environmental and biological sciences [11].

Fluorescence quenching is the process of reducing a substance’s fluorescence intensity. Quenching can occur through different mechanisms. Collisional quenching is the first type of quenching through deactivation of excited-state fluorophore when it comes into contact with any of the quenchers in the solution. In this case, the fluorophore diffuses into the quencher and returns to the ground state without emitting any photons. As a result, the molecules remain unchanged chemically [12]. A wide range of molecules can act as collisional quenchers, including amines, oxygen, halogens, and molecules with low electron densities like acrylamide.

The second type, known as static quenching, occurs when quenchers and fluorophores combine together to produce complexes that are not fluorescent. It occurs in the ground state and is unaffected by molecular collisions or diffusion [13].

The absorption spectra of the fluorophore can be used to differentiate between static and dynamic quenching. Since collisional quenching only affects the excited states of the fluorophores, no changes to the absorption spectra are expected, while the formation of ground-state complexes during static quenching frequently distorts the absorption spectra of the fluorophore [12].

Researchers have been eager to test the quenching theory using metal nanoparticles (NPs) because of their exceptional size-dependent optoelectronic characteristics.

Optical, catalytic, and electrical properties of silver nanoparticles (AgNPs) make them the most widely used.

When analytes interact with AgNPs, the quenching effect is either suppressed, elevating the intensity of fluorescence, or increased, diminishing fluorescence intensity [13].

Traditional quantification tools such as HPLC, UV–Vis spectrophotometry, and conventional fluorescence spectroscopy generally rely on spectral shifts, peak area integration, or absorbance changes, which can be subtle and require complex calibration and extensive sample preparation to eliminate matrix interferences. In contrast, plasmon-mediated quenching with silver nanoparticles (AgNPs) translates small molecular interactions directly into pronounced fluorescence intensity changes through localized surface plasmon resonance (LSPR)-assisted energy or electron transfer processes. This results in enhanced sensitivity, broader dynamic range, faster, and simpler workflows with less preprocessing than many traditional methods [14].

The goal of this research is to create a fluorescence quenching-based method that is rapid, easy, and sensitive for detecting the presence of Bosentan (BOS), an endothelin receptor antagonist that aids in the treatment of pulmonary hypertension in addition to its advantages in the treatment of COVID-19, in pharmaceutical formulations and spiked human plasma samples.

## Experimental

### Instrumentation

Shimadzu spectrofluorometer RF-6000 (Kyoto, Japan) with a slit width of 10.0 nm was utilized. Lab Solutions software (Rev. B.04.01, Shimadzu) was used for data acquisition, processing and instrument control. JEOL JEM-2100 high resolution transmission electron microscope. Vortex mixer (F20230176 ZX3, Alfa medical Westbury, China). Table-top centrifuge, Model PLC-012E (Gemmy Industrial Corp, Taiwan). Rotary evaporator equipped with vacuum pump (BUCHI Lab ortechnik AG, Switzerland).

### Materials

#### Chemicals and reagents

Ethanol, methanol, and acetonitrile of HPLC grade were purchased from Sigma-Aldrich (Cornell Lab, Cairo, Egypt). Deionized water (DW) was obtained from a MilliQ Plus system (Millipore Iberica, Spain). Isopropanol, and acetone were procured from El Nasr Company (Cairo, Egypt). Silver nanoparticles were purchased from Nano gate, (Cairo, Egypt). 4-tert-Butylcalix [8] arene (95%), hydroxyl propyl β cyclodextrin (HP-β-CD) and Beta-cyclodextrin (β -CD) (≥ 97%) were obtained from (Riedel-de Haën, Sigma- Aldrich, Germany). Sodium dodecyl sulphate (SDS), tween 80, triton X 100 and cetrimide were purchased from (ADWIC, Egypt).

Human plasma was acquired from VACSERA (Giza, Egypt) and kept at − 4 °C.

A Pulmiprove^®^ 62.5 mg Tablet (batch number 2031657) was procured from local market, Cairo, Egypt.

#### Pure standards

With great generosity, EVA Pharma (Cairo, Egypt) provided a certified BOS standard. Using the previously established HPLC method [15], its purity was determined to be 99.83% ± 0.45.

#### Standard solutions

##### Stock solutions

Stock solutions of all chemicals used in this study were prepared as follows:

- Bosentan (BOS): 0.01 g of BOS was accurately weighed and dissolved in 100 mL of methanol to prepare a 100.0 µg/mL stock solution.
- Silver nanoparticles (AgNPs): 0.01 g of AgNPs was dissolved in 100 mL of deionized water (DW) to prepare a 100.0 µg/mL stock solution.
- β-Cyclodextrin (β-CD) and Hydroxypropyl-β-cyclodextrin (HP-β-CD): 0.04 g of each compound was accurately weighed and transferred into separate 100 mL volumetric flasks, and the volume was made up to 100 mL with DW to prepare 400.0 µg/mL stock solutions.
- 4-tert-Butylcalix [8] arene: 0.046 g of 4-tert-Butylcalix [8] arene was dissolved in 100 mL of DMF to prepare a 460.0 µg/mL stock solution.
- Surfactants:
  - SDS and Cetrimide: 0.250 g of each surfactant was dissolved separately in 50 mL DW to prepare 0.5 g% (w/v) stock solutions.
  - Triton X-100 and Tween 80: 0.250 mL of each surfactant was dissolved in 50 mL DW to prepare 0.5 v/v% stock solutions.

All stock solutions were stored at appropriate conditions until use and further diluted to the desired working concentrations as needed for experiments.

##### BOS working solution

Working standard solution of BOS having concentration of 1.0 µg/mL was prepared by transferring 1.0 mL of BOS stock solution to a 100.0 mL volumetric flask and completing to the final volume with methanol.

### Procedure

#### Spectral characteristics of AgNPs alone and in the presence of BOS

A 10.0 mL volumetric flask was filled with 0.1 mL of AgNPs from its stock solution, then ethanol was added to complete the volume. To attain a concentration of 1.0 µg/mL of AgNPs and 0.1 µg/mL of BOS, 0.1 mL of AgNPs from its stock solution and 1.0 mL of BOS from its working solution were added to a second 10.0 mL volumetric flask. Ethanol was then added to complete the final volume. Ethanol was used as a blank to measure the excitation and emission fluorescence spectra of both solutions at 402.0 nm and 466.4 nm, respectively.

#### The impact of experimental conditions on AgNPs fluorescence quenching by BOS

The effects of various factors, including AgNPs concentration, solvent type, micellar media, and complexing agents, on AgNPs fluorescence quenching by BOS were investigated.

##### Effect of different AgNPs concentrations on AgNPs fluorescence quenching by BOS

A set of 10.0 mL volumetric flasks was used to precisely transfer 0.03, 0.05, 0.07, 0.09, 0.1, 0.13, and 0.15 mL of its stock solution to create various concentrations of AgNPs. Ethanol was then added to complete the final volume. An additional batch of 10.0 mL volumetric flasks was made using the same process, adding 1.0 mL of BOS working solution to each flask. Every time, 466.4 nm was used to measure the emission fluorescence spectrum. The software eliminated the blank spectra created with ethanol from the matching spectra. After obtaining values of F° (fluorescence intensities of AgNPs in the absence of BOS) and F (fluorescence intensities of AgNPs in presence of BOS) for each concentration level, the relative fluorescence intensity (F^°^/ F) was plotted against the concentration of AgNPs.

##### Effect of solvents on AgNPs fluorescence quenching by BOS

A variety of solvents, such as methanol, DW, ethanol, isopropanol, acetone and acetonitrile, were employed. After transferring 0.1 mL of the AgNPs stock solution to a set of 10.0 mL volumetric flasks, the remaining volume was made up using the solvents listed above. An additional batch of 10.0 mL volumetric flasks was made using the same process, adding 1.0 mL of BOS working solution to each flask. Every time, 466.4 nm was used to measure the emission fluorescence spectrum. The blank spectra that were prepared by the mentioned solvents were subtracted by the software from the corresponding spectra. The change in (F^°^/ F) was recorded each time.

##### **Effect of micellar media on AgNPs fluorescence quenching by BOS**

A series of 10.0 mL volumetric flasks were filled with 0.1 mL of the AgNPs stock solution. Next, 1.0 mL of SDS, triton X100, cetrimide, or tween 80 stock solutions were added. Finally, ethanol was used to make up the final volume. Using the same procedure, a second batch of 10.0 mL volumetric flasks was prepared, adding 1.0 mL of BOS working solution to each flask. Each time, the emission fluorescence spectrum was measured at 466.4 nm. The blank was prepared with the surfactant only and the final volume was completed with ethanol. The change in (F^°^/ F) was recorded each time.

##### **Effect of complexing agents on AgNPs fluorescence quenching by BOS**

A volume of 0.1 mL of AgNPs stock solution was transferred to a series of 10.0 mL volumetric flasks, then 1.0 mL 4- tert-Butyl calix [8] arene stock solution, 1.0 mL β-CD stock solution (400.0 µg/mL) or 1.0 mL HP-β-CD (400.0 µg/mL) were added, and then the final volume was completed with ethanol. Using the same procedure, a second batch of 10.0 mL volumetric flasks was made, adding 1.0 mL of BOS working solution to each flask. Each time, the emission fluorescence spectrum was measured at 466.4 nm. The blank was prepared with the complexing agents only and the final volume was completed with ethanol. The change in (F^°^/ F) was recorded each time.

#### Method validation

##### Linearity

After carefully transferring aliquots of BOS working solution into a series of 10.0 mL volumetric flasks and adding 0.1 mL of AgNPs stock solution to each flask, the volume was filled to the mark with ethanol to produce BOS concentrations ranging from 0.02 to 0.12 µg/mL. Following excitation at 402.0 nm, the intensity of the emission fluorescence was measured at 466.4 nm. The regression equation was calculated, and a calibration curve was created between the fluorescence intensity ratio (F^°^ /F) and the corresponding concentration of BOS.

##### Accuracy

By performing the previously described procedure under 2.3.3.1, five distinct BOS concentrations (0.03, 0.05, 0.07, 0.09, and 0.11 µg/mL) were generated and measured. The standard deviation and percentage recoveries were computed after the concentrations were determined using the corresponding regression equation.

##### Precision

Using the previously described procedure under 2.3.3.1, three replicate of various BOS concentrations (0.03, 0.07, and 0.11 µg/mL) were generated and determined on the same day as well as on three separate days. The %RSD were computed after the concentrations were determined using the corresponding regression equation.

##### LOD and LOQ

The following formulae [16] were used to calculate LOD and LOQ:

$${\mathrm{LOD}}\,=\,{\mathrm{3}}.{\mathrm{3}} \times \sigma /{\mathrm{S}}$$

$${\mathrm{LOQ}}\,=\,10 \times \sigma /{\mathrm{S}}$$

Where S is the slope of the calibration curve for the drug under study, and σ is the standard deviation of the intercept.

### Application

#### Analysis of BOS in pharmaceutical tablet formulation

Ten Pulmiprove ® 62.5 mg tablets were accurately weighed, yielding a mean total weight of 0.140 gm per tablet. The tablets were then finely pulverized, and an accurately weighed portion of the powder, equivalent to 0.001 gm of BOS, was transferred to a 100.0 mL volumetric flask. The powder was dissolved in ethanol, and the final volume was adjusted to 100.0 mL with ethanol to prepare a solution with a concentration of 10.0 µg/mL.

Volumetric flask was shaken for approximately 10.0 min, and the contents were filtered through Whatman filter paper no. 42. Using a series of 10.0 mL volumetric flasks, aliquots of this filtered solution were precisely transferred to prepare different concentrations of (0.03, 0.055, 0.075, and 0.095 µg/mL). Next, 0.1 mL of AgNPs stock solution was added to each flask, and finally the final volume was completed by ethanol. Each time, the emission fluorescence spectrum was measured at 466.4 nm. The corresponding regression equation was used to calculate the found concentrations. To check the validity of the proposed method, standard addition technique was applied by analyzing the formulation spiked with different concentrations of BOS standards (0.03, 0.055, and 0.08 µg/mL), and the percentage recoveries were then calculated.

#### Analysis of BOS in spiked human plasma

Blank human plasma was added to a number of falcon tubes, and the tubes were then spiked with the appropriate quantities of BOS working solution to provide three distinct concentrations (0.02, 0.034, and 0.06 µg/mL), including the highest plasma concentration (C_max_) after receiving a single 3.0 mg dose [17]. Next, tubes were vortexed. Plasma protein was then precipitated by adding 4.0 ml of methanol. Tubes were subsequently centrifuged at a speed of 6000 rpm for twenty minutes. The supernatant was dried by evaporation and then reconstituted in 10.0 mL volumetric flask with ethanol, after that 0.1 ml AgNPs was added. The final volume was completed by ethanol. Emission fluorescence spectra were recorded at 466.4 nm and (F) was calculated. The same procedures were used to create plasma samples, but no drug was spiked in order to determine (F^°^).

## Results and discussion

A new, sensitive, eco-friendly, precise, and accurate spectrofluorometric probe was created for the first time to measure BOS in pharmaceutical dosage forms and human plasma.

**Fig. 2:**
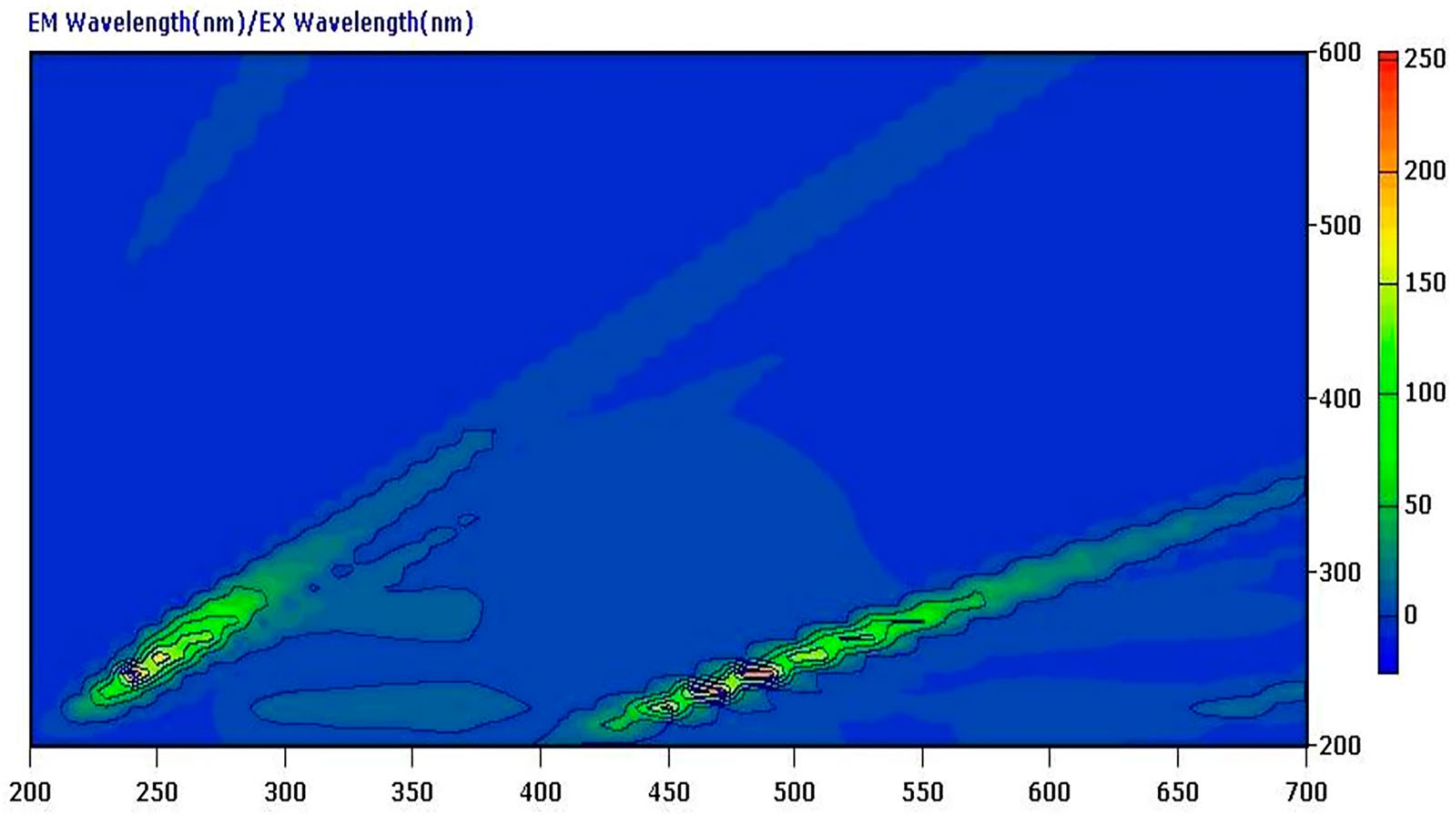
3D fluorescence scan spectrum of 0.1 µg/mL BOS at excitation range (200.0–600.0 nm), and emission range (200.0–700.0 nm)

**Fig. 3:**
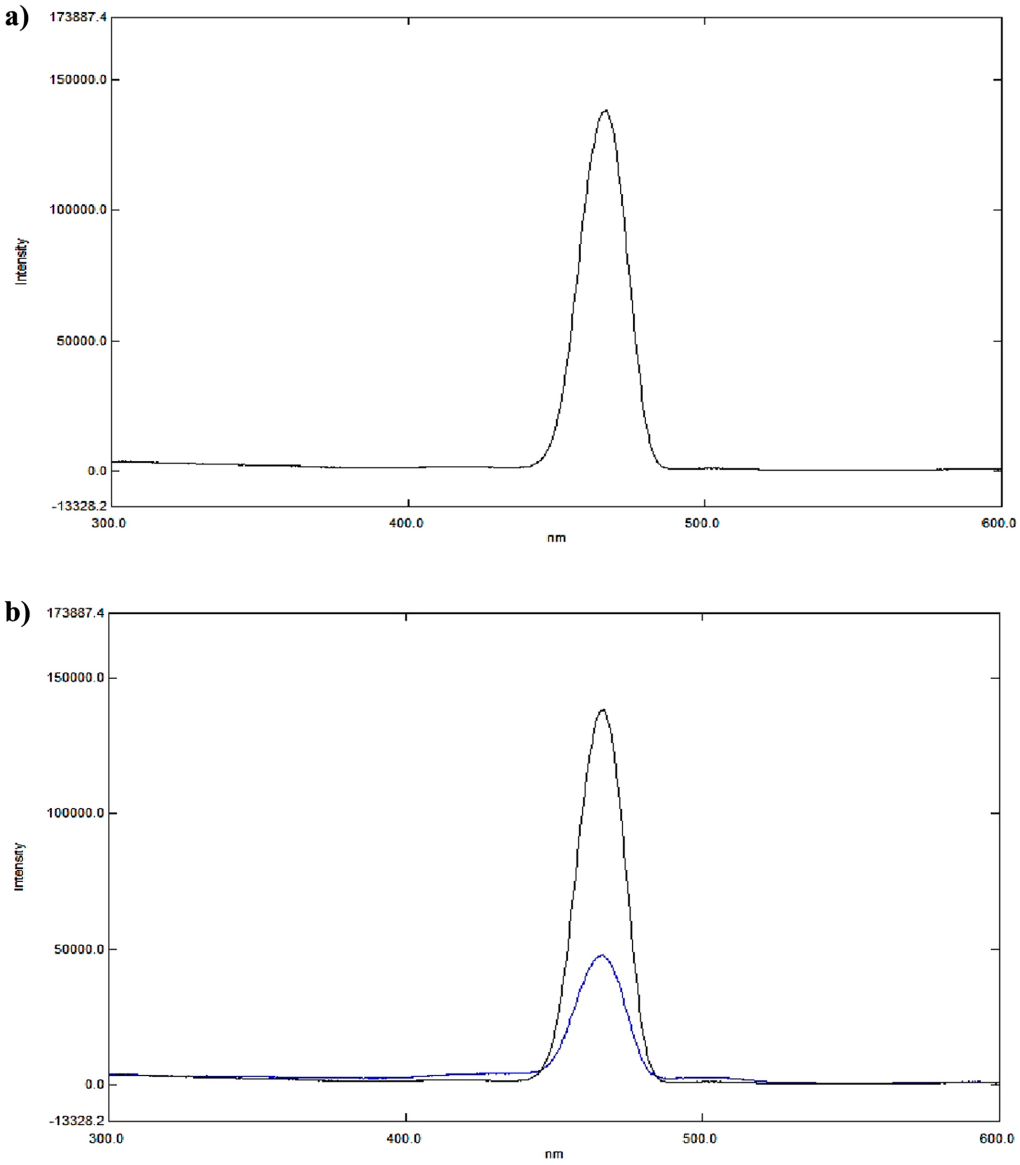
**a** Emission spectrum of 1.0 µg/mL AgNPs at 466.4 nm after excitation at 402.0 nm. **b** Quenching of 1.0 µg/mL AgNPs fluorescence intensity with the addition of 0.1 µg/mL BOS at 466.4 nm

Although BOS lacks natural fluorescence as shown in its 3D spectrofluorometric scan and displayed in Fig. 2, it may quench AgNPs emission fluorescence [18] via a dynamic (collisional) quenching technique because its structure contains several oxygen and amine groups as illustrated in Fig. 3a, and Fig. 3b. The morphology and size of AgNPs were assessed by a transmission electron microscope (TEM). Fig.S1 shows TEM micrograph, illustrating the spherical nature of AgNPs, and an average particle size of approx. 30.0 nm.

**Fig. 4:**
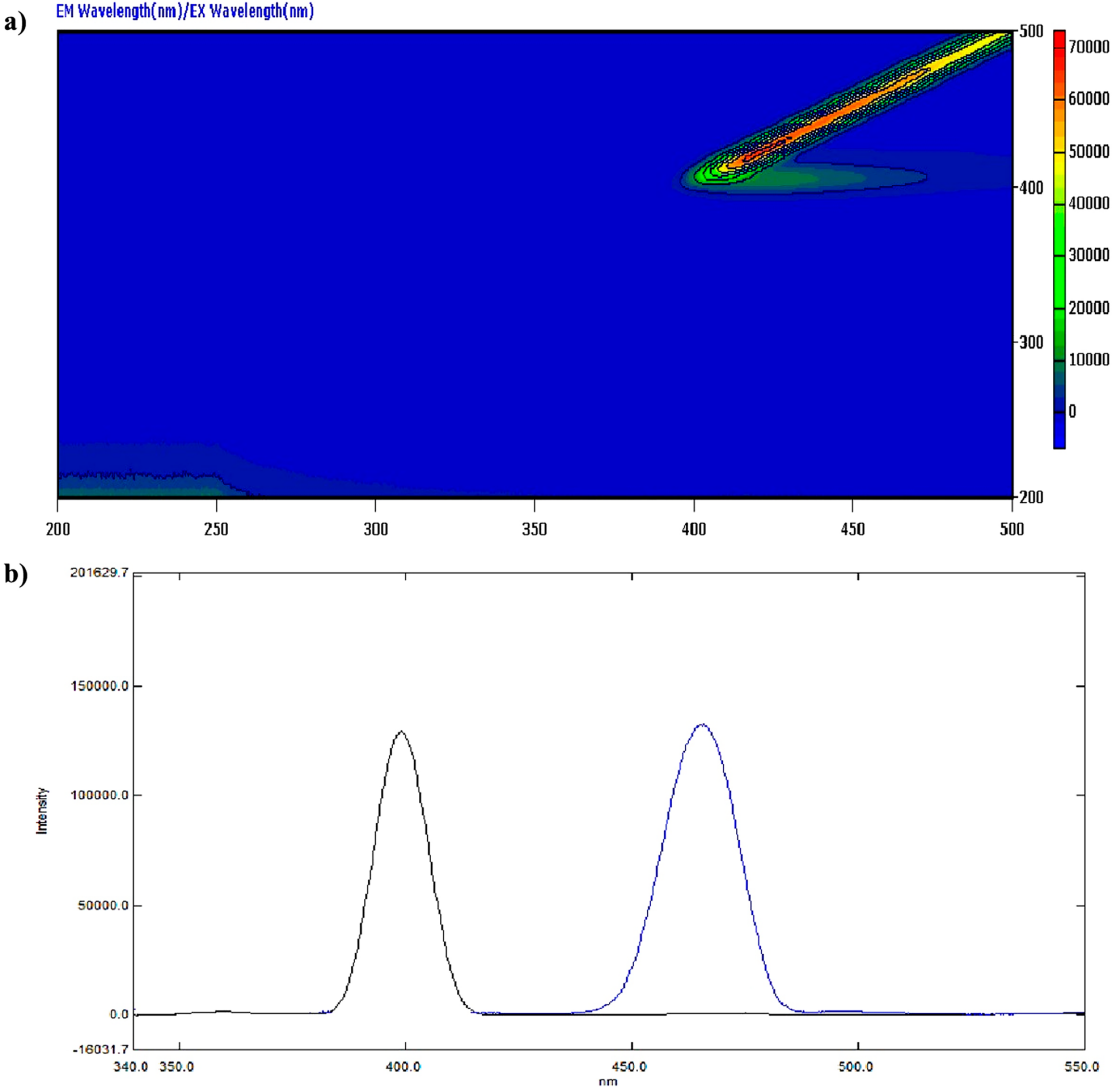
**a** 3D fluorescence scan spectrum of 1.0 µg/mL AgNPs at excitation range (200.0–500.0 nm), and emission range (200.0–500.0 nm). **b**. Excitation-Emission spectra of 1.0 µg/mL AgNPs at 402.0 nm, and 466.4 nm, respectively

3D spectrofluorometric scan, and excitation – emission spectra of AgNPs at 402.0 nm, and 466.4 nm are illustrated in Fig. 4a, and Fig. 4b, respectively. While Fig. S2. (a) and (b) show Emission spectra of ethanol (blank) and of 1.0 µg/mL AgNPs in ethanol after excitation at 402.0 nm.

The Stern–Volmer equation:

$$ {\mathrm{F}}^\circ /{\mathrm{F}}{\mkern 1mu} = {\mkern 1mu} {\mathrm{1}}{\mkern 1mu} + {\mkern 1mu} {\mathrm{K}}_{{\mathrm{D}}} \left[ {\mathrm{Q}} \right] $$

F^°^: Fluorescence intensities of AgNPs in the absence of BOS.

F: Fluorescence intensities of AgNPs in the presence of BOS.

K_D_: the Stern-Volmer constant.

[Q]: concentration of BOS.

**Fig. 5:**
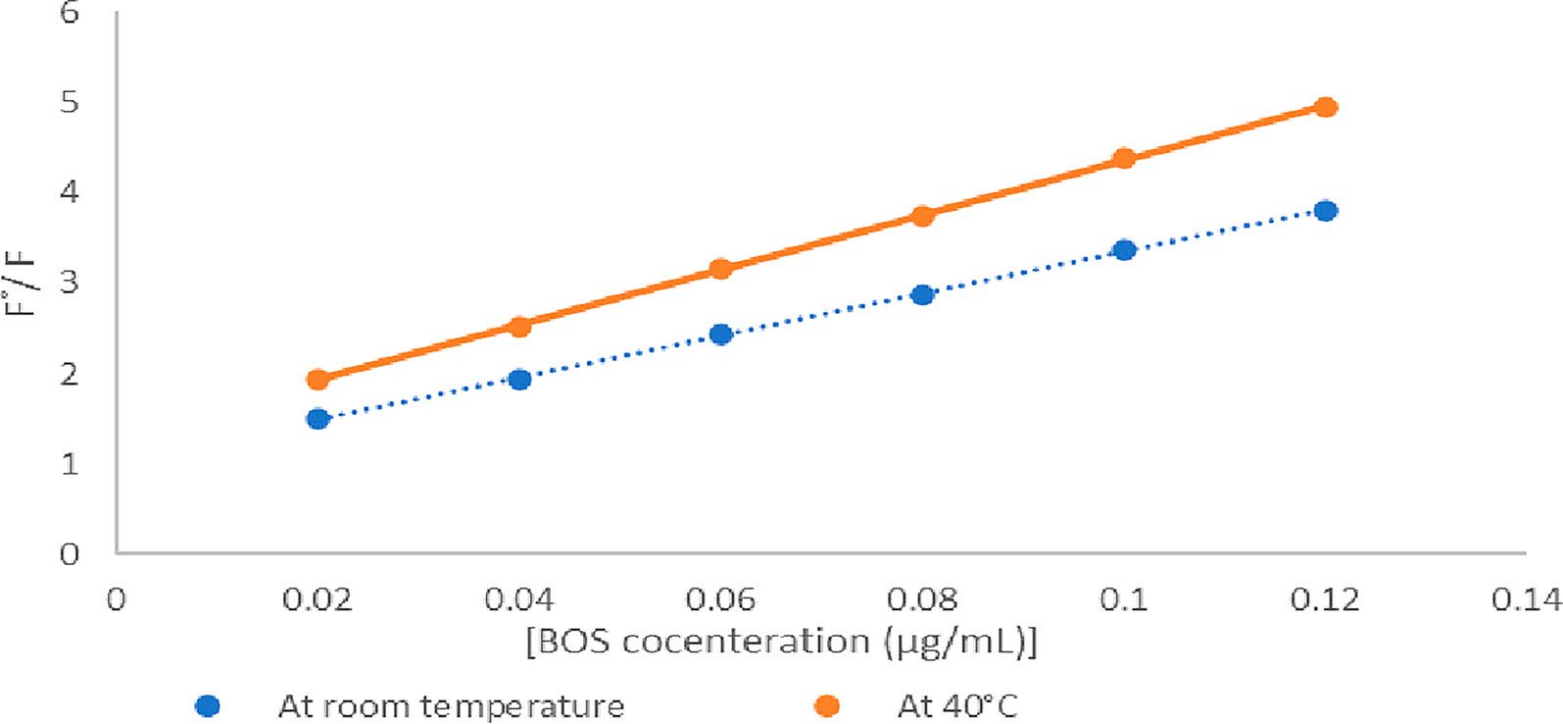
Stern-Volmer plots of AgNPs-BOS mixtures at different temperatures

The above equation shows the intermolecular deactivation as a function of the quencher molecule concentration, which can be used to characterize the dynamic quenching mechanism [19]. Because dynamic quenching relies on diffusion, temperature has an impact on it [20]. So, to understand the nature of the quenching process, studying temperature dependence is required. Figure 5 shows an increase in quenching effect at higher temperatures (40 °C) with an increased quenching constant K_D_ value that is indicated by the slope of temperature plot, which suggests that the mechanism of fluorescence quenching is by collision quenching.

### The impact of experimental conditions on AgNPs fluorescence quenching by BOS

#### Effect of different AgNPs concentrations

FigureS3 illustrates that the concentration of 1.0 µg/mL of AgNPs has the greatest (F^°^ /F) value compared to lower concentrations. Meanwhile, higher concentrations have no additional effect.

#### Effect of different solvents

As illustrated in Fig.S4, ethanol was determined to be the best solvent for this investigation among all the solvents examined, followed by DW and methanol. The lowest (F^°^ /F) was found for acetone.

#### Effect of micellar media

The possible impact of various micellar media on (F^°^ /F) was investigated. Surfactants were used, although at concentrations greater than the critical micellar concentration, fluorescence intensity was suppressed by using various micellar medium as illustrated in Fig.S5. As a result, the suggested approach did not involve the use of surfactants.

#### Effect of complexing agents

The effect of various complexing agents on (F^°^/F) was investigated. It was found that the fluorescence peak of AgNPs disappeared on adding complexing agents. So, using complexing agents did not add any benefits to this study.

### Method validation

The created approach was validated in compliance with ICH guidelines for method validation [21].

#### Linearity

When the concentration of BOS increased, the quenching effect of BOS on AgNPs increased over a concentration range of (0.02–0.12 µg/mL) as shown in Fig.S6a, and thus the (F^°^ /F) ratio increased. The calibration curve shown in Fig.S6b illustrates the linear relationship between the (F^°^ /F) ratio and the corresponding BOS concentration in the range of (0.02–0.12 µg/mL).

Table 1 provides a summary of the calibration curve’s analytical data, which includes linear range, slope, intercept, and correlation coefficient.

**Table 1:** Results of assay validation for the analysis of pure BOS samples using the suggested AgNPs fluorescence quenching method

Parameters	BOS
Concentration Range (µg/mL)	0.02–0.12
Slope	23.2611
Intercept	1.0253
Correlation coefficient (r)	0.9998
Accuracy (Mean ± SD) *	100.10 ± 1.28
Repeatability (% RSD) *	0.16
Intermediate precision (%RSD) *	0.62
LOD (µg/mL)	0.0159
LOQ (µg/mL)	0.02

* Average of three replicates

#### Accuracy

Three replicates of five BOS concentration levels were used to assess the accuracy of the suggested approach. As seen in Table 2, the recovery percentages fell between 98.33% and 101.11%, demonstrating the accuracy of this approach.

**Table 2:** Accuracy results for the analysis of pure BOS samples using the suggested AgNPs fluorescence quenching method

Claimed concentration (µg/mL)	Found concentration (µg/mL)	% Recovery*
0.03	0.0295	98.33 ± 1.63
0.05	0.0505	101.00 ± 1.43
0.07	0.0694	99.14 ± 1.47
0.09	0.091	101.11 ± 1.58
0.11	0.111	100.91 ± 1.01
Mean ± SD		100.10 ± 1.28

*Average of three replicates

#### Precision

The precision of the proposed method was evaluated in terms of repeatability and intermediate precision. Repeatability was assessed by analyzing three replicates of BOS at different concentration levels on the same day, yielding a %RSD of 0.16. Intermediate precision was evaluated over three consecutive days, resulting in a %RSD of 0.62. These low %RSD values indicate that the method is highly precise, reproducible, and suitable for the accurate determination of BOS as illustrated in Table 1.

#### LOD and LOQ

Table 1 presents the results of determining the LOQ and LOD for the suggested approach. These values indicate that the method is highly sensitive, as it can reliably detect and accurately quantify BOS at very low concentrations. Considering that the typical BOS concentrations in pharmaceutical dosage forms and plasma samples are several orders of magnitude higher than these limits.

### Application

#### Analysis of BOS in pharmaceutical tablet formulation

The developed probe was successfully used with good percentage recoveries and low standard deviation values to determine BOS in pharmaceutical formulation. An additional measure of method validity (standard addition) was used. Table 3 provides a summary of the results, showing acceptable recovery percentage values with low SD values confirming the validity of this approach.

**Table 3:** Analysis of BOS in its pharmaceutical formulation using the suggested AgNPs fluorescence quenching method and application of standard addition technique

Pulmiprove® 62.5 mg tablets			Standard addition technique			
Claimed concentration (µg/mL)	Found concentration (µg/mL)	% Recovery*	Taken (Dosage form concentration) (µg/mL)	Pure added (µg/mL)	Pure Found (µg/mL)	% Recovery*
0.03	0.0294	98.00	0.03	0.03	0.0300	100.00
0.055	0.0548	99.64		0.055	0.0558	101.45
0.075	0.0763	101.73		0.08	0.0807	101.88
0.095	0.0935	98.42				
Mean ± SD		99.45 ± 1.67	Mean ± SD			100.11 ± 0.99

*Average of three replicates

#### Analysis of BOS in spiked human plasma

The new method was applied successfully to quantify BOS in spiked human plasma following protein precipitation using methanol, yielding good percentage recoveries and low standard deviation value. Table 4 provides a summary of the results.

**Table 4:** Analysis of BOS in spiked human plasma using the suggested AgNPs fluorescence quenching method

Spiked concentration (µg/mL)	Found concentration (µg/mL)	%Recovery*
0.02	0.0204	102.00
0.034	0.0346	101.76
0.06	0.0591	98.50
Mean ± SD		100.75 ± 1.96

*Average of three replicates

### Statistical analysis

A statistical analysis was performed to compare the outcomes of the suggested approach with those of the reported one [15]. The calculated t and F values were found to be smaller than the tabulated ones, indicating no statistically significant difference. Table 5 presents the results, which indicate that the suggested approach is determined to be exact and accurate.

**Table 5:** Statistical comparison between the proposed method and the reported method for the determination of BOS

Parameter	Reported method* [15]	Proposed method
Mean	99.83	100.10
SD	0.45	1.28
Variance	0.20	1.64
n	3.0	5.0
Student t test (2.447)		0.34
F (19.247)		8.20

*Inertsil C8 column (5 µ, 15 cm x 4.6 mm) followed by a guard column CLC ODS (4 cm x 4.6 mm, i.d.) was used for chromatographic separation by isocratic elution. Acetate buffer (pH 5.5) and acetonitrile in the ratio of 20:80 (v/v) were used as mobile phase with a flow of 1.0 mL/min

### Greenness assessment of the proposed spectrofluorimetric method

Green analytical chemistry (GAC) is an important concept that is steadily gaining attention as a result of increased environmental consciousness since it might lessen the potentially negative environmental effects that analytical techniques may have [22]. Thus, our aim was the development of green analytical method for the analysis of the studied drug. The greenness of the developed method was assessed using AGREE greenness assessment tool, along with Analytical Eco-scale (AES).

#### Analytical eco-scale (AES)

The analytical eco-scale [23] is calculated by deducting penalty points from a base of 100 for each analytical method component. In conformity with its standards, the method that is ideally green scores an eco-scale of 100, the excellent green method scores an eco-scale of more than 75, and the acceptable green method scores an eco-scale of more than 50 [24]. When the method yields an eco-scale score of less than 50, it is believed to be an inadequately green analytical method.

The analytical eco-scale was calculated for the proposed method in Table 6 showing that the developed method is an excellent green method with a high eco-scale score of 80.0.

#### Analytical greenness metric tool (AGREE)

AGREE methodology applies all 12 green analytical chemistry (GAC) principles, producing a result that is both easily interpretable and informative among all the greenness rating methods [25]. If the AGREE analytical score for drug analysis is higher than 0.75, the analytical procedure is considered green. Furthermore, a score of 0.50 means that the drug analysis method is acceptable in terms of its greenness. From a greenness perspective, a score below 0.50 indicates that the suggested analytical procedure is insufficiently green.

AGREE software was used to assess the greenness of the proposed method as shown in Table 6 proving the greenness of the developed method with a score of 0.68.

**Table 6:** Greenness assessment of BOS proposed spectrofluorimetric method with analytical Eco- scale and AGREE software

Hazard	Analytical Eco-Scale (Penalty points)
Reagents	
AgNPs	4.0
Methanol	6.0
Ethanol	4.0
Instruments	
Energy (< 0.1 kWh per sample)	0
Occupational hazard	0
Waste	6.0
Total penalty points	20.0
Analytical Eco-Scale total score	80.0
AGREE software	

### Comparison of the proposed method to other conventional methods for BOS determination

The analytical performance of the proposed method for the determination of bosentan (BOS) was compared with several conventional methods reported in the literature. As summarized in Table 7, the proposed method demonstrated superior analytical quality parameters, particularly in terms of sensitivity, linearity, and precision. The method showed a low limit of detection (LOD = 0.0159 µg/mL) and limit of quantification (LOQ = 0.02 µg/mL), along with excellent precision, where repeatability and intermediate precision values were below 1% RSD.

**Table 7:** Comparison between different analytical methods for BOS determination

Method		Linearity (µg/mL)	LOD / LOQ (µg/mL)	Accuracy (%/Recovery)	Precision (%RSD)	Advantages	Disadvantages	References
The proposed method		0.02–0.120	LOD 0.0159, LOQ 0.02	100.10 ± 1.28	Repeatability 0.16; Intermediate 0.62	✔ High sensitivity & good precision ✔ Excellent linearity at low concentration range ✔ High accuracy		[Current method]
RP-HPLC UV (bulk/tablets)		20–120	LOD 0.55, LOQ 1.71	98–102%	< 2%	✔ Applicable for routine QC	✗ higher LOD/LOQ values ✗ Higher cost	[26]
RP-HPLC UV		0.005–0.07	LOD 0.002, LOQ 0.007	~ 98.6%	< 2%	✔ Good precision and recovery ✔High sensitivity	✗ Higher cost	[27]
Voltammetric method	LSV	5–40	LOD 1.6, LOQ 4.8	100.80%	Repeatability 1.72–5.34; Intermediate 1.65–4.24	✔ Fast, and simple	✗ Higher LOD/LOQ values	[28]
	SWV	5–35	LOD 0.9, LOQ 2.7	101.85%	Repeatability 3.25–4.42; Intermediate 3.89–4.36			
	DPV	5–35	LOD 0.3, LOQ 2.9	100.85%	Repeatability 2.60–4.22; Intermediate 3.66–5.25			
Stability-Indicating RP-HPLC UV		0.25–20	LOD 0.1, LOQ 0.25	< 2.7% RE	< 3%	✔ Useful for impurities	✗ Higher LOD/LOQ values than new method ✗ Higher cost	[29]

### Conclusion

In this study, a novel eco-friendly fluorescence quenching–based analytical method was successfully developed and validated for the sensitive determination of BOS. The proposed method fulfills our objective by providing excellent analytical performance with a markedly low limit of detection (LOD = 0.0159 µg/mL) and limit of quantification (LOQ = 0.02 µg/mL), alongside a narrow linear range optimized for low-concentration analysis and outstanding precision (%RSD < 1%).

The novelty of the proposed method lies in the effective utilization of fluorescence quenching as a detection strategy, which enables high sensitivity without the need for complex instrumentation, extensive sample preparation, or time-consuming analytical procedures. Compared with several conventional methods reported in the literature, including UV-spectrophotometric, voltammetric, and routine RP-HPLC techniques, the developed method demonstrates superior sensitivity and improved analytical reliability, particularly for trace-level quantification and at low cost. These advantages represent a significant improvement over other reported conventional approaches that are limited by higher detection limits or higher cost.

Importantly, the applicability of the proposed method was successfully demonstrated in pharmaceutical formulations and spiked human plasma samples, confirming its suitability for both quality control and bioanalytical purposes. Overall, the developed fluorescence quenching–based method offers a robust, precise, and sensitive analytical tool for BOS determination, and a potential value for routine analysis and future clinical or pharmaceutical applications.

## Supplementary Information

Below is the link to the electronic supplementary material.

Supplementary Material 1.

## Data Availability

All data generated or analyzed during this study are included in this published article [and its supplementary information files].

## References

[1] Sysol JR, Machado RF. Classification and pathophysiology of pulmonary hypertension. Contin Cardiol Educ. 2018;4:2–12. 10.1002/cce2.71.

[2] Shi Y, Wang Y, Shao C, Huang J, Gan J, Huang X, Bucci E, Piacentini M, Ippolito G, Melino G. COVID-19 infection: the perspectives on immune responses. Cell Death Differ. 2020;27:1451–4. 10.1038/s41418-020-0530-3. 32205856 10.1038/s41418-020-0530-3 PMC7091918

[3] Khaled S, et al. Bosentan and pulmonary hypertension caused by COVID-19: a pilot randomized double-blind clinical study. Curr Vas Pharmacol. 2024;22:437–46. 10.0000/pmid38874033. 10.2174/0115701611299843240607061547 38874033

[4] Trabik YA, Ismail RA, Ayad MF, Hussein LA, Mahmoud AM. Microfabricated potentiometric sensor based on a carbon nanotube transducer layer for selective Bosentan determination. Rev Anal Chem. 2024;43. 10.1515/revac-2023-0071.

[5] Sajedi-Amin S, Assadpour-Zeynali K, Panahi-Azar V, Kebriaeezadeh A, Khoubnasabjafari M, Ansarin K, Jouyban-Gharamaleki V, Jouyban A. Spectroscopic analysis of Bosentan in biological samples after a liquid–liquid Microextraction. BioImpacts. 2015;5:191–7. 10.15171/bi.2015.28. 26929923 10.15171/bi.2015.28 PMC4769789

[6] Pavani B, Harshavardhan B, Sindhu Priya G, Sharanya K. Kanchan. Method development and validation of Bosentan by UV spectrophotometric method. J Drug Deliv Ther. 2018;1:34–8. 10.0000/uvbosentan2018.

[7] Khalighi Z, Ghaneialvar H, Soltani A, Khorshidi A, Karimi E, Moayeri A, Abbasi N, Tahmasebi M, Aidy A. Simple determination of Bosentan in plasma samples by reversed-phase HPLC. Avicenna J Med Biotechnol. 2024;16:104–10. 38618512 10.18502/ajmb.v16i2.14861 PMC11007376

[8] Yaman ME, Atila A, Kadıoğlu Y. Stability-indicating RP-HPLC method development and validation for Bosentan in pharmaceutical formulations. J Turk Chem Soc A. 2022;9:505–12. 10.18596/jotcsa.956110.

[9] Jatczak M, Sidoryk K, Kossykowska M, Łuniewski W, Zagrodzka J, Lipiec-Abramska E. Development and validation of a UHPLC-UV method for in-process control of Bosentan monohydrate synthesis. Chromatographia. 2016;79:1131–41. 10.1007/s10337-016-3124-y. 27616782 10.1007/s10337-016-3124-y PMC4995228

[10] Kamel MZ, Yamani HZ, Hussein LA, Trabik YA. First fluorescence method for monitoring the doping stimulant drug solriamfetol in plasma and urine. Microchem J. 2024;207:111930. 10.1016/j.microc.2024.111930.

[11] Li W, Wang L, Tang H, Cao D. Diketopyrrolopyrrole-based fluorescent probes for detection and bioimaging. Dyes Pigm. 2019;162:934–50. 10.1016/j.dyepig.2018.11.023.

[12] Šachl R, Amaro M, editors. Fluorescence spectroscopy and microscopy in biology. Cham: Springer; 2023. 10.1007/978-3-031-30362-3.

[13] Abd Elhaleem SM, Elsebaei F, Shalan S, Belal F. Effect of silver nanoparticles on fluorescence intensity of bambuterol and Terbutaline using FRET. J Fluoresc. 2023;33:1717–25. 10.1007/s10895-023-03182-7. 36826730 10.1007/s10895-023-03182-7 PMC10539440

[14] Salari SM, Khani H, Rostami A, et al. Silver nanoprism-based fluorescence sensors for ultrasensitive detection. RSC Adv. 2025;15:841–59. 10.1039/D4RA08469A.

[15] Siddappa K, Hanamshetty P. Development and validation of an HPLC method for Bosentan in pure and formulated forms. Der Pharm Lett. 2016;8:404–11. 10.0000/derpharmlett2016.

[16] Harris DC. Quantitative chemical analysis. 9th ed. New York: W.H. Freeman; 2016. 10.0000/quantchem2016.

[17] Dingemanse J, van Giersbergen PLM. Clinical Pharmacology of bosentan, a dual endothelin receptor antagonist. Clin Pharmacokinet. 2004;43:1089–115. 10.2165/00003088-200443150-00003. 15568889 10.2165/00003088-200443150-00003

[18] Rodríguez-Santana P, Jiménez-Abizanda AI, Hernández-Creus A, Jiménez-Moreno F. Synthesis and characterization of ag nanoparticles and interaction with Fe. J Lumin. 2017;190:207–14. 10.1016/j.jlumin.2017.05.020.

[19] Gehlen MH. The centenary of the Stern–Volmer equation of fluorescence quenching. J Photochem Photobiol C. 2020;42:100338. 10.1016/j.jphotochemrev.2019.100338.

[20] Hley J, Manikova P. Fluorescent sensors. In: fundamentals of sensor technology. Woodhead Publishing. 2023;147–61. 10.1016/B978-0-323-88431-0.00022-3.

[21] International Council for Harmonisation (ICH). ICH Q2(R2): validation of analytical procedures. Geneva; 2024. 10.0000/ICHQ2R2.

[22] Płotka-Wasylka J, Namieśnik J. Green analytical chemistry: past, present perspectives. Springer; 2019. 10.1007/978-981-13-9105-7.

[23] Yin L, Yu L, Guo Y, Wang C, Ge Y, Zheng X, Zhang Y, Zhang J, Shi M. Green analytical chemistry metrics. J Pharm Anal. 2024;14:101013. 10.1016/j.jpha.2024.101013. 39759968 10.1016/j.jpha.2024.101013 PMC11697060

[24] Fahmy NM, Abdullatif HA, Michael AM, Ayad MF, Trabik YA. Eco-friendly spectrophotometric methods resolving overlapping spectra. J AOAC Int. 2022;105:1234–46. 10.1093/jaoacint/qsac058. 35543495 10.1093/jaoacint/qsac058

[25] Pena F, Wojnowski W, Tobiszewski M. Analytical greenness metric approach software. Anal Chem. 2020;92:10076–82. 10.1021/acs.analchem.0c0188. 32538619 10.1021/acs.analchem.0c01887 PMC7588019

[26] Saidulu P, Masthanamma SK, Anitha Kumari V. New validated RP-HPLC method for the determination of Bosentan in bulk and dosage form. Res J Pharm Technol. 2015;8:549–53. 10.5958/0974-360X.2015.00091.8.

[27] Muralidharan S, Raja Kumar J. Simple Estimation of Bosentan in tablet formulation by RP-HPLC. Am J Anal Chem. 2012;3:715–8. 10.4236/ajac.2012.311095.

[28] Atila A, Yilmaz B. Determination of Bosentan in pharmaceutical preparations by linear sweep, square wave and differential pulse voltammetry methods. Iran J Pharm Res. 2015;14:443–51. 10.22037/ijpr.2015.1649. 25901151 PMC4403060

[29] Yaman Y, Atıla A, Kadioglu Y. Stability indicating RP-HPLC method development and validation for Bosentan in pharmaceutical formulations. J Turkish Chem Soc Chem. 2022;9:505–12. 10.18596/jotcsa.956110.

