# Supplementary figures and images for: Silver nanoparticles-based green fluorescent probe for determination of Bosentan in pharmaceutical formulation and spiked plasma samples
Source: BMC Chem. 2026 Feb 21;20(1):44. doi: 10.1186/s13065-026-01737-w (PMC12930978; doi:10.1186/s13065-026-01737-w)

**Supplementary materials**

**
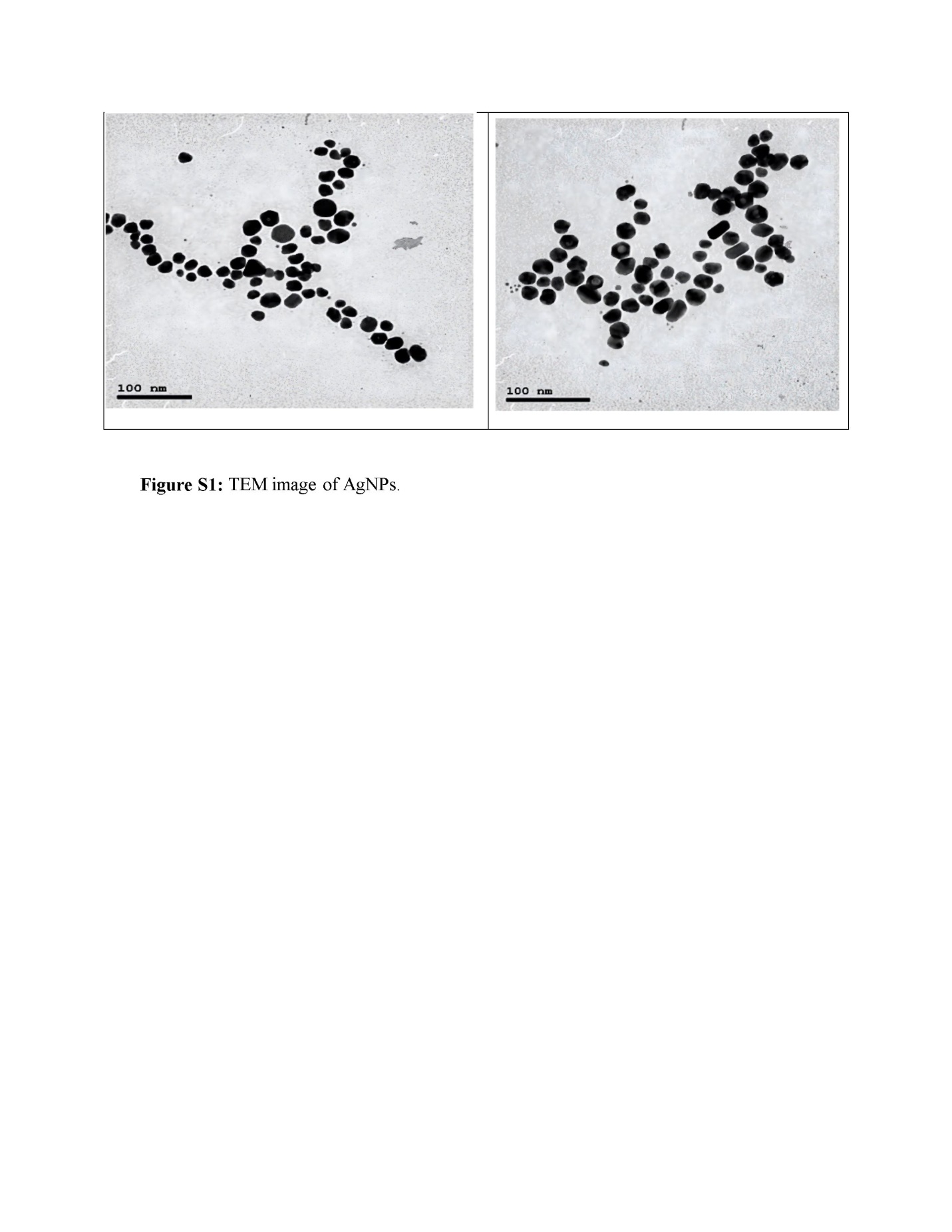
**

**
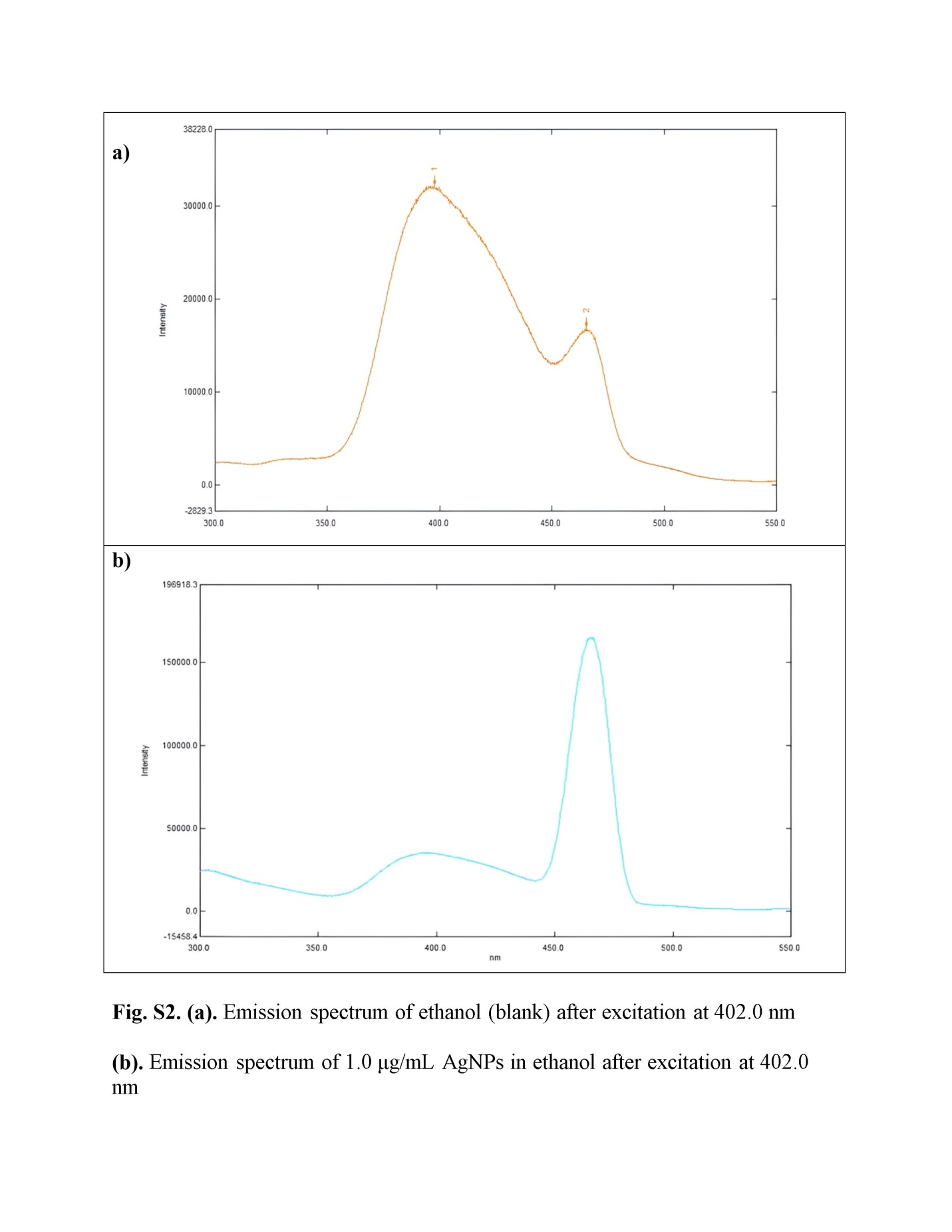
**


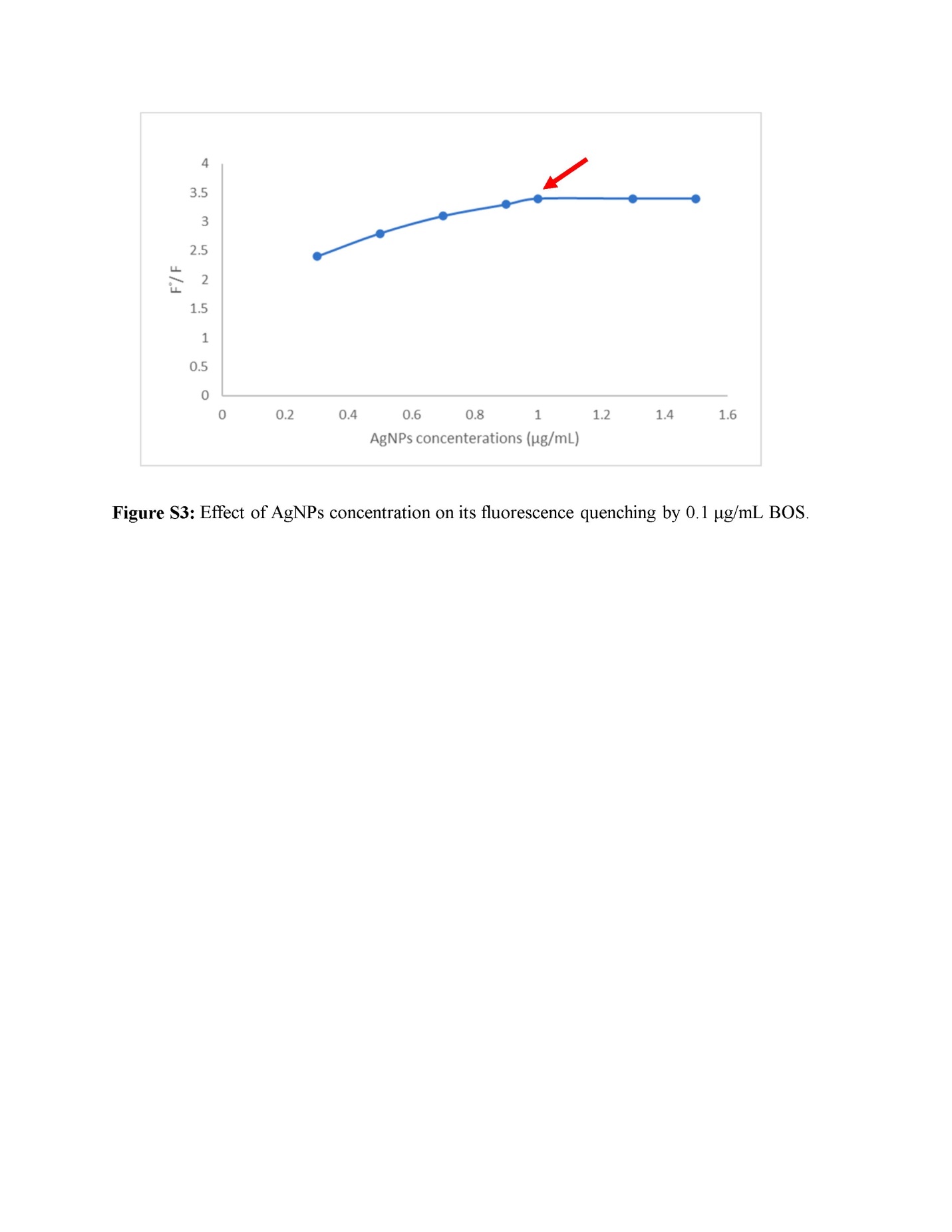


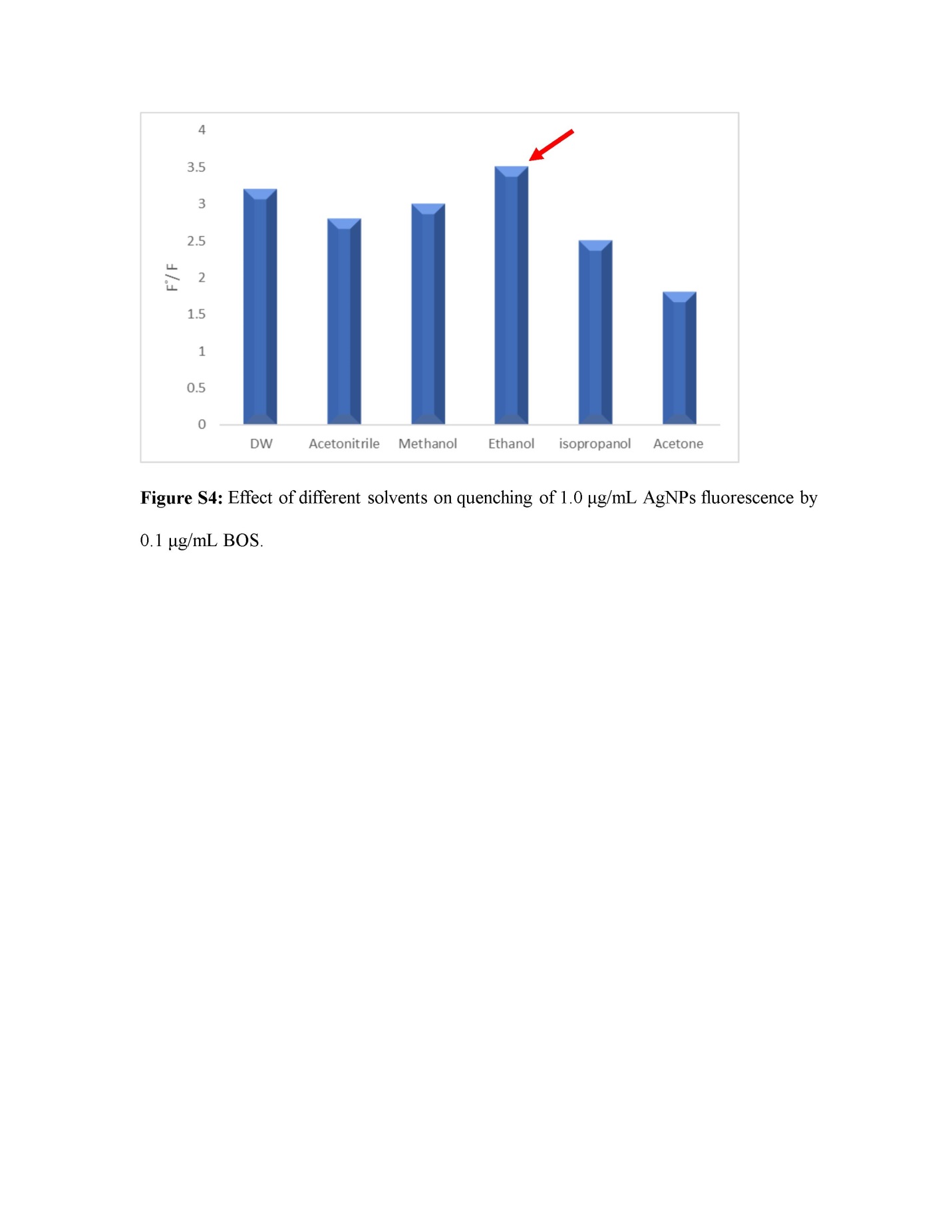


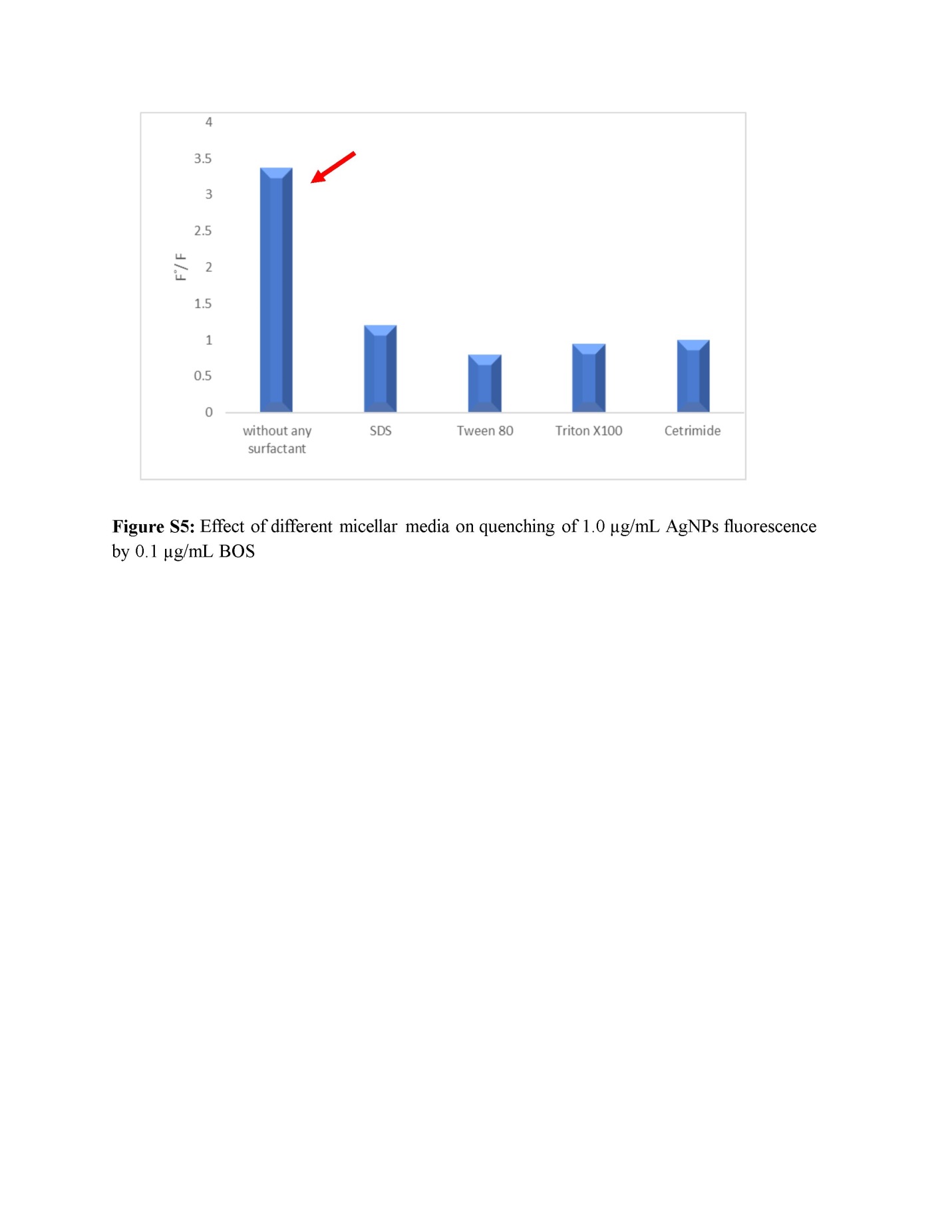


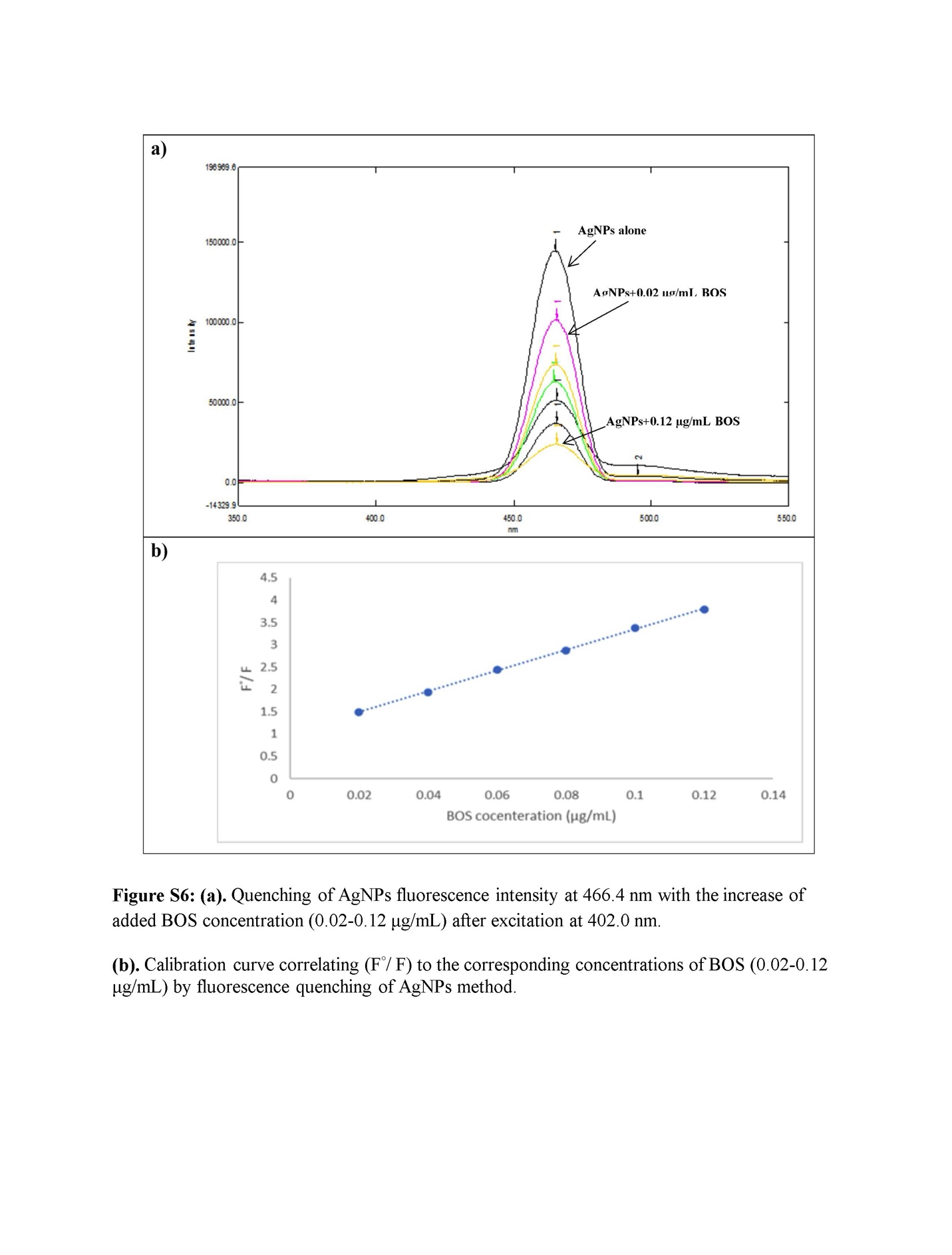

Supplement: Supplementary file 1 — Supplementary Material 1. [file 13065_2026_1737_MOESM1_ESM.docx]
